# Structural and biochemical studies of sulphotransferase 18 from *Arabidopsis thaliana* explain its substrate specificity and reaction mechanism

**DOI:** 10.1038/s41598-017-04539-2

**Published:** 2017-06-23

**Authors:** Felix Hirschmann, Florian Krause, Petra Baruch, Igor Chizhov, Jonathan Wolf Mueller, Dietmar J. Manstein, Jutta Papenbrock, Roman Fedorov

**Affiliations:** 1 Institute of Botany, Leibniz University Hannover, Herrenhäuserstr. 2, D-30419 Hannover, Germany; 2 Institute for Biophysical Chemistry, Hannover Medical School, Carl-Neuberg-Strasse 1, D-30625 Hannover, Germany; 3 Research Division for Structural Biochemistry, Hannover Medical School, Carl-Neuberg-Strasse 1, D-30625 Hannover, Germany; 4 Institute of Metabolism and Systems Research (IMSR), University of Birmingham, Birmingham, B15 2TT UK; 5 Centre for Endocrinology, Diabetes and Metabolism (CEDAM), Birmingham Health Partners, Birmingham, B15 2TH UK

## Abstract

Sulphotransferases are a diverse group of enzymes catalysing the transfer of a sulfuryl group from 3′-phosphoadenosine 5′-phosphosulphate (PAPS) to a broad range of secondary metabolites. They exist in all kingdoms of life. In *Arabidopsis thaliana* (L.) Heynh. twenty-two sulphotransferase (SOT) isoforms were identified. Three of those are involved in glucosinolate (Gl) biosynthesis, glycosylated sulphur-containing aldoximes containing chemically different side chains, whose break-down products are involved in stress response against herbivores, pathogens, and abiotic stress. To explain the differences in substrate specificity of desulpho (ds)-Gl SOTs and to understand the reaction mechanism of plant SOTs, we determined the first high-resolution crystal structure of the plant ds-Gl SOT AtSOT18 in complex with 3′-phosphoadenosine 5′-phosphate (PAP) alone and together with the Gl sinigrin. These new structural insights into the determination of substrate specificity were complemented by mutagenesis studies. The structure of AtSOT18 invigorates the similarity between plant and mammalian sulphotransferases, which illustrates the evolutionary conservation of this multifunctional enzyme family. We identified the essential residues for substrate binding and catalysis and demonstrated that the catalytic mechanism is conserved between human and plant enzymes. Our study indicates that the loop-gating mechanism is likely to be a source of the substrate specificity in plants.

## Introduction

**Figure 1:**
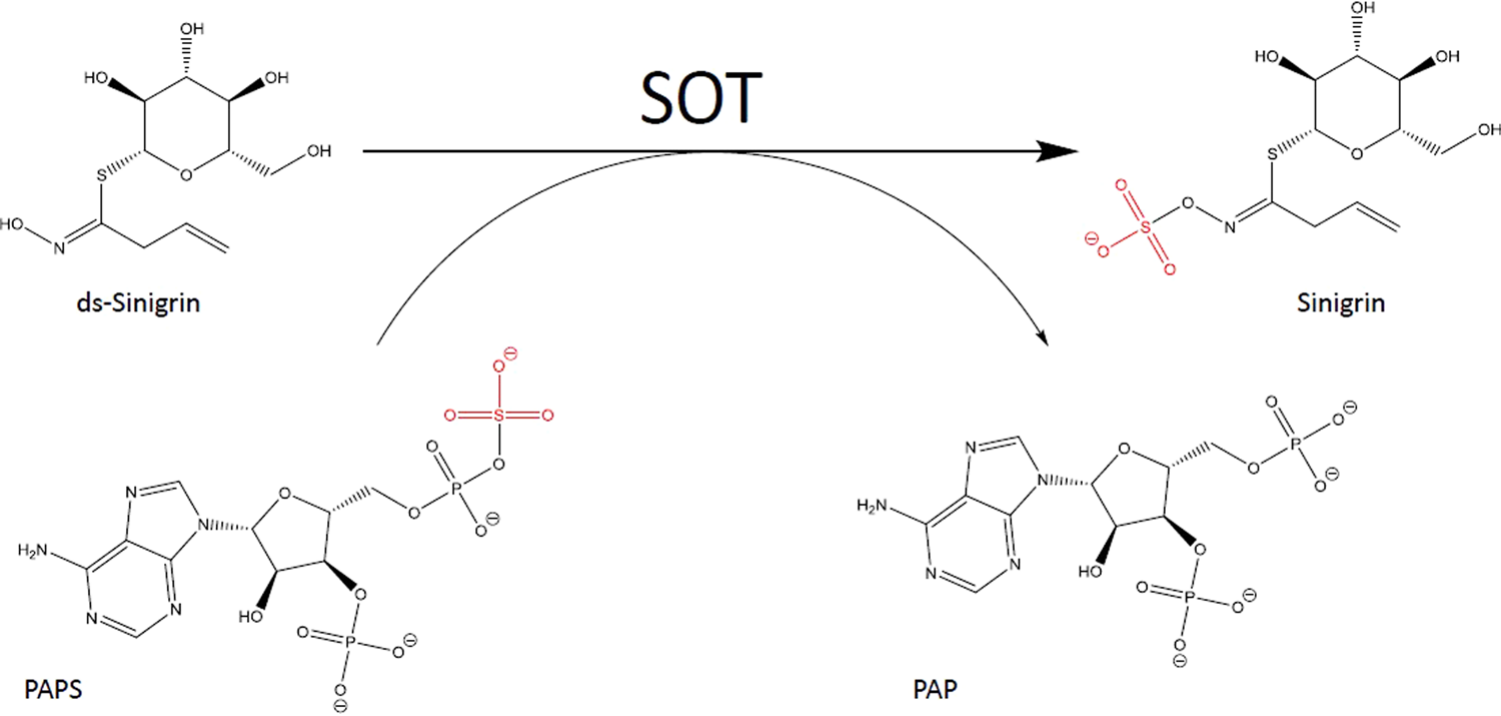
AtSOT18 catalysed reaction. The red coloured sulphate ester with carbon-oxygen-sulphur bonding (R-O-S-O_3_^−^) is transferred from PAPS to the hydroxyl group of the ds-Gl sinigrin.

Sulphotransferases (SOTs or SULTs) (EC 2.8.2.-) can be found in all organisms analysed so far. They catalyse the transfer of a sulfuryl group from the co-substrate 3′-phospho- adenosine 5′-phosphosulphate (PAPS) to a hydroxyl group of various substrates. In plants, sulphated compounds act as hormones, secondary metabolites in stress defense and probably serve as a reservoir for sulphur^1^. The role of plant SOTs in the sulphation of desulpho-glucosinolates (ds-Gl) is of particular interest (Fig. 1), as they are important secondary metabolites and their break-down products are involved in defence against herbivores, pathogens, and abiotic stress in the plant order Brassicales^2^.

For humans, Gl degradation products play a role as flavour compounds from numerous cabbage, radish and mustard species. As an example, allyl isothiocyanate accounts for the spiciness of horseradish and mustard^3^. Some Gl degradation products may have negative characteristics, e.g. goitrogenicity^4^, and can be toxic to humans^5^. On the other hand, the anti-cancerogenic activity of some Gls, such as sulphoraphan (1-isothiocyanato-4-methylsulphinyl-butan Gl) from broccoli and cabbage, is of high interest for the development of new medical treatments^6, 7^.

**Figure 2:**
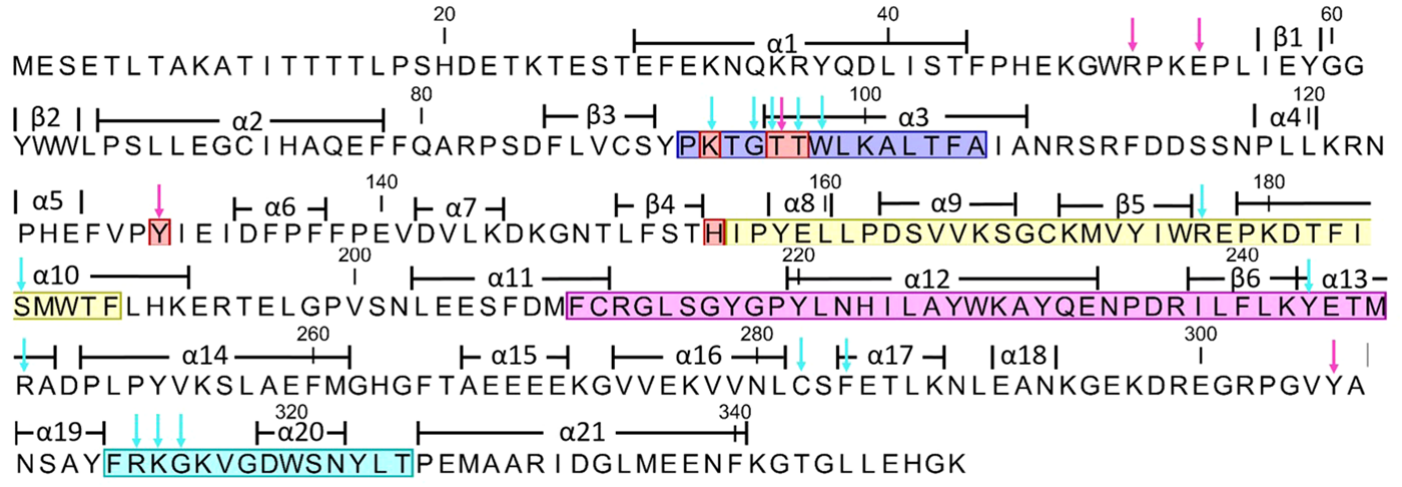
AtSOT18 amino acid sequence. Conserved regions as defined by^8^ (region I: blue; region II: yellow; region III: magenta: region IV: cyan), catalytic residues (red), PAP binding residues (cyan arrows), sinigrin binding residues (magenta arrows).

Most sulphotransferases are characterized by four conserved regions (I–IV)^8^ on the level of amino acid sequence (Fig. 2), including a highly conserved catalytic histidine at the beginning of region II^9^. For plant SOTs the functions of these regions have not yet been identified, but at least two have been suggested to be involved in PAPS binding^10^.

In *Arabidopsis thaliana* the three SOTs AtSOT16, AtSOT17 and AtSOT18 exclusively catalyse the transfer of a sulfuryl group to different ds-Gls^11–13^. All three enzymes are localized in the cytoplasm and their expression pattern tested under several conditions is similar^11^. These three AtSOTs share at least 72% sequence identity but differ remarkably in their substrate specificity. *In vitro* enzyme assays revealed a striking preference of AtSOT16 for the indolic ds-Gl indol-3-yl-methyl Gl (I3M). AtSOT17 showed an increased specific activity with long-chained ds-Gls derived from methionine. AtSOT18 preferred the long-chain ds-Gls 7-methylthioheptyl Gl and 8-methylthiooctyl Gl (8MTO), that are also derived from methionine^14^. Furthermore, we could recently demonstrate that even AtSOT18 enzymes from different ecotypes vary in their substrate specificities^15^. However, the molecular basis for the observed substrate specificity of ds-Gl SOTs is not well understood.

The actual reaction mechanism of sulphotransferases is still under debate. Eukaryotic soluble SULTs follow a sequential mechanism, in either a specific or independent order^14, 16^, while eukaryotic membrane-associated^17^ and bacterial SULTs^18^ follow a ping-pong mechanism. For the well-studied human sulphotransferases, the order of the nucleophilic substitution is not determined. Kinetic isotope effect studies suggested an S_N_1 mechanism^16^, while crystal structures with PAPS and substrate suggest an S_N_2-like inline displacement^19^.

In addition to the basic characterization of the two-substrate reaction mechanism, a better understanding of the ds-Gl SOTs structure-function relationship and the mechanisms of substrate specificity could contribute to the development of strategies for manipulating and optimizing the Gl content and composition of crop plants in the Brassicaceae family. Thus, the therapeutic and biotechnological potential of Gl-containing plant species as nutraceuticals, and as a source of anti-cancerogenic and antimicrobial compounds could be fully exploited.

So far, SULT structures from *Mus musculus*, *Homo sapiens*, and several prokaryotes have been published^9, 20^. Furthermore, the structure of the apo-form of *A. thaliana* SOT12 has been solved^17^. Structurally, all soluble sulphotransferase enzymes share a common fold consisting of four central β-strands surrounded by α-helices. Three flexible loops, gating the substrate binding site were reported to influence substrate specificity^18, 20^. The conformational properties of these loops in the apo-state of AtSOT12 remain unclear, due to the lack of structural information^17^.

Here we report crystal structures of the AtSOT18 binary complex with PAP and a ternary complex with PAP and sinigrin. To gain insights into the reaction kinetics of AtSOT18, we performed inhibition/activation assays with PAP and PAPS, using two-dimensional fit of enzymatic titration data. The residues important for catalysis were identified by a combination of structural, mutagenesis and kinetic studies. Analysis of the substrate binding site indicates that the substrate specificity in plant SOTs is controlled outside of the active centre, most likely by the gating mechanism utilizing three functional loops around the active site pocket.

## Results

### The structure of AtSOT18

**Figure 3:**
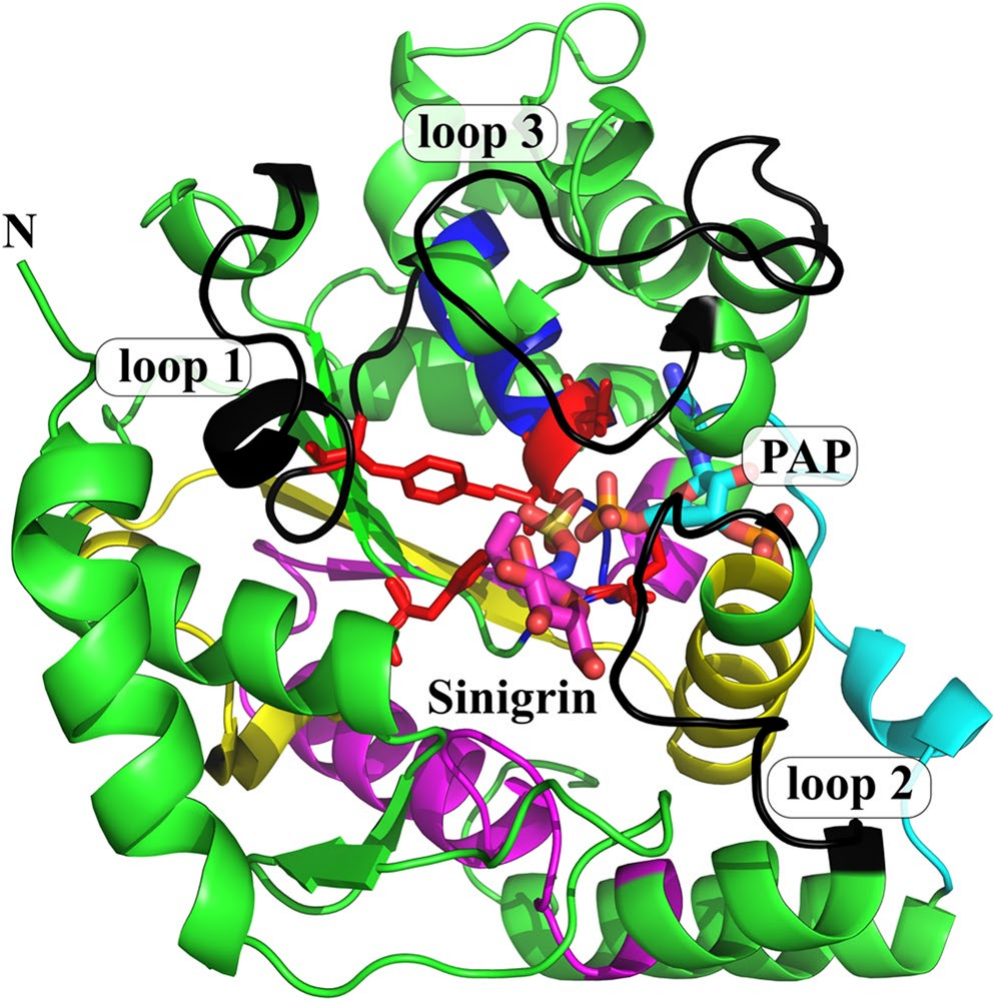
Overall view of AtSOT18 from two perspectives bound with sinigrin (magenta sticks) and PAP (cyan sticks). Indicated are the four conserved regions (region I: blue; region II: yellow; region III: magenta: region IV: cyan), the three flexible loop regions (black), the catalytic residues (red) and proline 136 (orange). The upper view shows the three flexible loops gating the sinigrin binding site. The view below shows how two of the four typical β-strands are formed by the conserved regions II and III.

**Figure 4:**
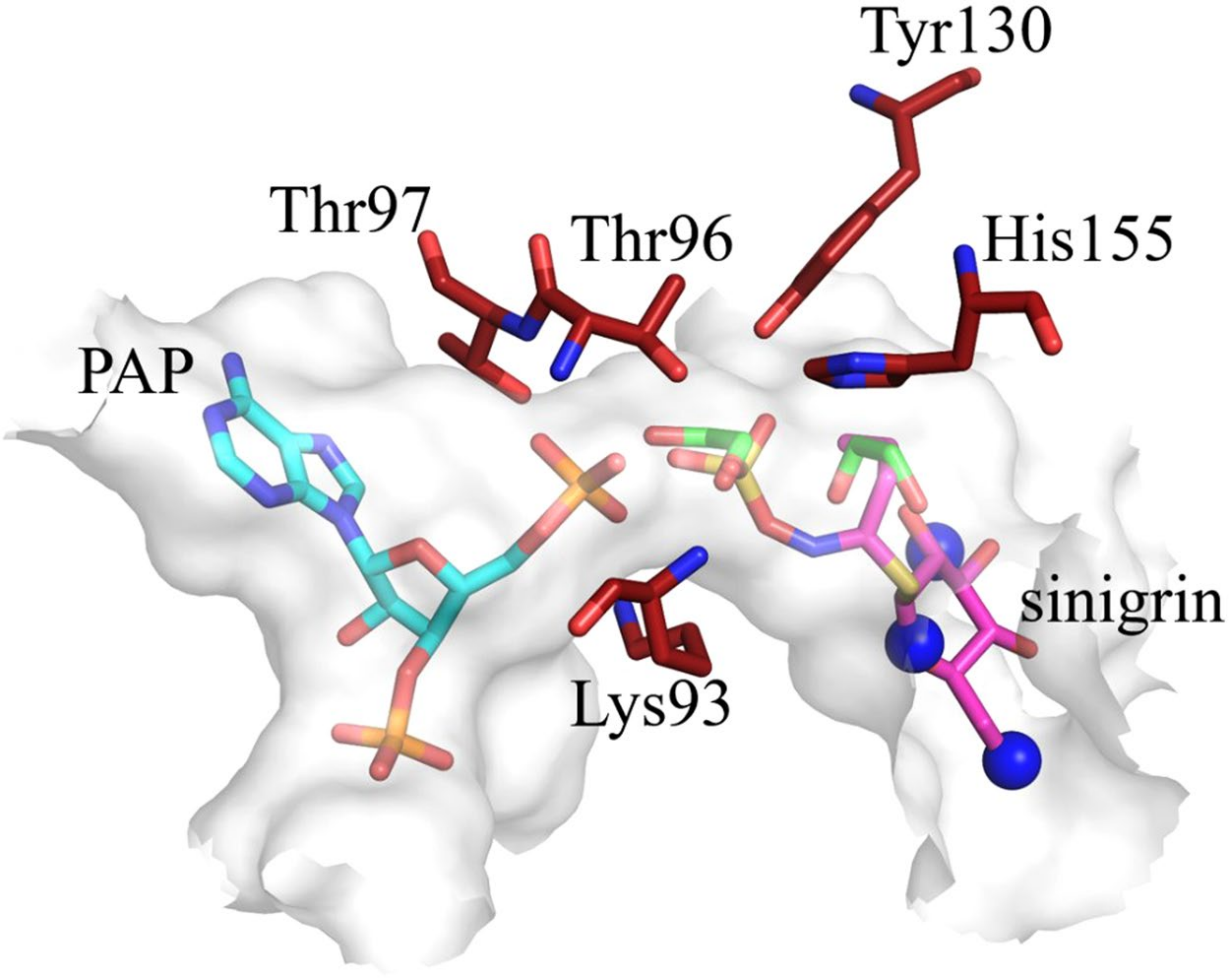
Close up of the binding site with residues of the catalytic centre. Catalytic residues (red) with bound sinigrin (magenta), PAP (cyan) and protein surface (light grey). Several atomic positions occupied by the polar groups of sinigrin in the AtSOT18•PAP•sinigrin complex, including the sulphate moiety, are filled with solvent molecules (water: blue spheres; ethane-1,2-diol: green sticks) in the AtSOT18•PAP complex structure.

**Figure 5:**
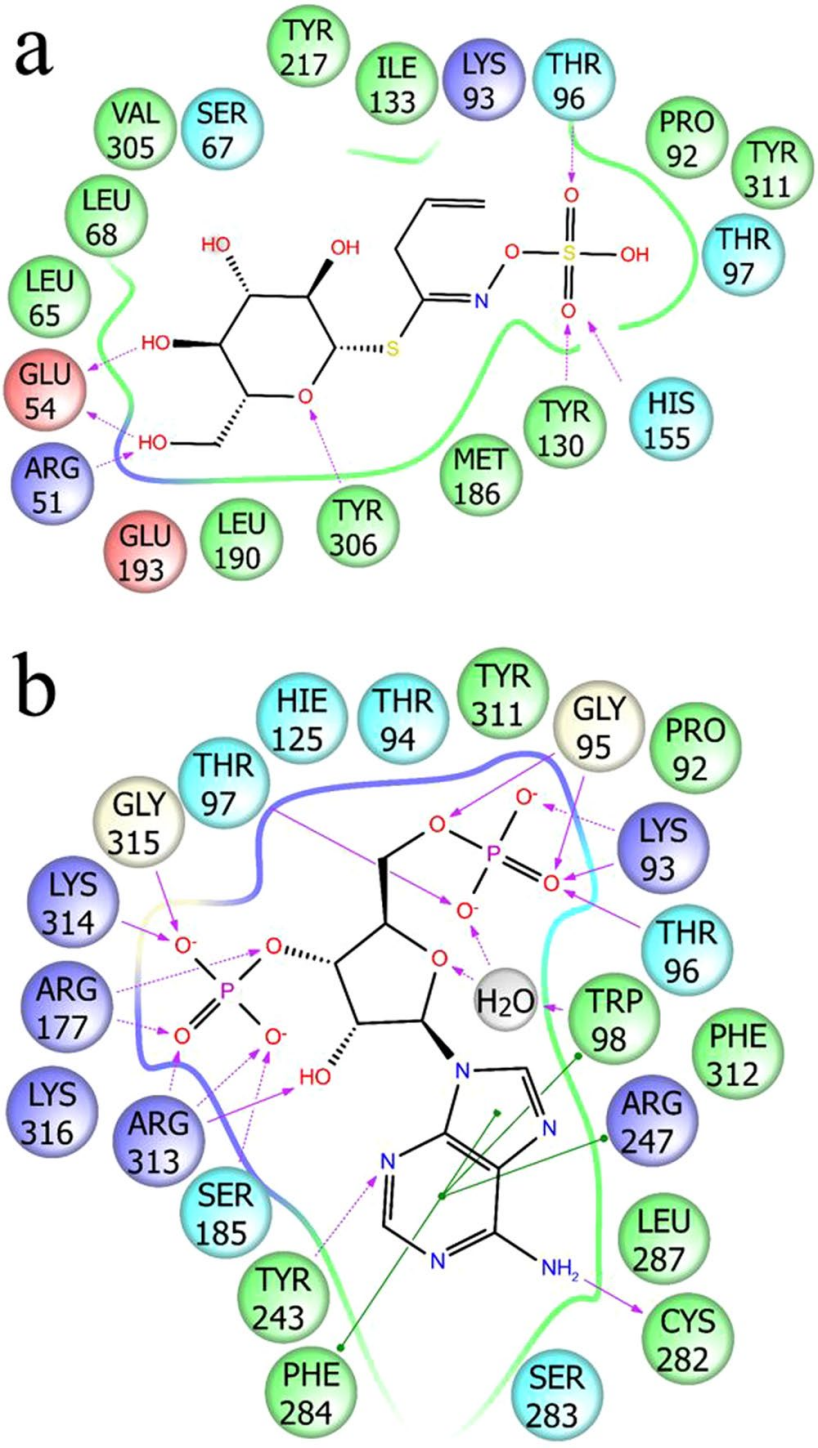
Ligand binding sites (**a**) sinigrin binding site and (**b**) PAP binding site with and π-π stacking (green lines) interaction and hydrogen bonds to residue backbone (purple arrows) and side chains (dotted purple arrows).

To address ds-Gl SOT specificity and catalysis, the high-resolution X-ray structures of AtSOT18 in complex with the co-product PAP alone (PDB ID: 5MEK) and together with the product Gl sinigrin (PDB ID: 5MEX) were solved (Fig. 3). The overall fold of AtSOT18 is similar to the previously described mammalian enzymes^20, 21^. The fold consists of four central β-strands forming the characteristic backbone, surrounded by 12 α-helices and two additional smaller β-strands (Fig. 3). Also the three typical flexible loops gating the acceptor binding site could be identified. The highly conserved His155 is localized in the catalytic centre, where it makes a strong hydrogen bond (2.5 Å) with the sulphate moiety of sinigrin (Figs 4 and 5a).

According to DALI^22^, our SOT18 complexes have the highest structural identity to apo-AtSOT12 (41% amino acid identity)^17^, followed by human SULT1A1^23^ and SULT1A3^24^. Interestingly, the dimerization motif KxxxTVxxxE, which is conserved in all human SULTs^20^ is neither present in AtSOT18 nor in any other *A. thaliana* SOT.

The high similarity of AtSOT18 structure to mammalian sulphotransferases illustrates the evolutionary conservation of this multifunctional enzyme family. Hence, the functional divergence of plant SOTs could be explained by the role of the unique residues in the individual isoforms.

### Crystallographic analysis of substrate binding in the AtSOT18 complexes

Towards a better understanding of the specific ds-Gl SOT structural characteristics, we analysed the substrate binding sites in complexes with sinigrin and PAP and PAP alone. The comparison revealed no major structural differences (Fig. S1). The two models could be overlaid with a root mean square deviation (RMSD) of 0.187 Å. Only the Met186 side chain undergoes a major conformational change upon sinigrin binding **(**Fig. S2 **)**. Both AtSOT18 complex structures showed two openings to the active site cavity. One is located directly at the acceptor site as an entry for the substrates and has dimensions of approximately 14 × 9 Å. Another opening has smaller dimensions of 10 × 7 Å and is located close to the adenyl group of PAP.

Sinigrin binding (Fig. S3 **)** is facilitated by hydrogen bonding with residues Arg51, Glu54, Thr96, Tyr130, His155 and Tyr306 (Fig. 5a). The guanidinium group of Arg51 interacts with the 6’-hydroxyl group of the glucopyranose of the Gl. The carboxyl group of Glu54 interacts with the 4′- and the 6′-hydroxyl groups. The hydroxyl of Tyr306 forms a hydrogen bond with the oxygen in the glucopyranose ring. The sulphate moiety is stabilized by hydrogen bonds with Thr96, Tyr130 and His155. In this positioning the sulphate and phosphate groups of sinigrin and PAP, respectively, are located within the hydrogen bond distance from each other. Several atomic positions occupied by the polar groups of sinigrin in the AtSOT18•PAP•sinigrin complex, including the sulphate moiety, are filled with solvent molecules in the AtSOT18•PAP complex structure (Fig. 4).

The quality of the electron density allowed an unambiguous determination of the position and conformation of the PAP (Fig. S4). PAP is deeply embedded within the structure and stabilized by several hydrogen bonds and π-π stacking with surrounding amino acids. The four oxygen atoms of the 3′-phosphate group form six hydrogen bonds with the side chains of Arg177, Ser185, Arg313, and the main chain of Lys314 and Gly315 (Fig. 5b). The oxygen atoms of the 5′-phosphate group make six hydrogen bonds to the side chains of Lys93, the main chain of Gly95, and both the main and the side chains of Thr96 and Thr97. Arg313 formed a hydrogen bond with the hydroxyl group at the 2′-carbon of PAP. The adenyl moiety of PAP is stabilized by hydrogen bonds with Cys282 and Tyr243, the stacking interaction with Trp98, and the hydrophobic contacts with Phe284 and aliphatic groups of Arg247. Out of 14 residues that contact PAP directly, 11 are within the highly conserved regions I-IV (except Arg247, Cys282, and Phe284), while only a single amino acid (Thr96) of the five sinigrin-binding residues is in there (Fig. 3). In the AtSOT18•PAP complex, the main conformation of Met186 is oriented away from the acceptor site (Fig. S2). Upon sinigrin binding, the methionine side chain sometimes turns toward the acceptor, providing an additional stabilization to its hydrophobic moiety.

In summary, structural analysis of the PAP and sinigrin binding revealed that the Gl sinigrin is rather loosely bound compared to the tightly bound PAP. Furthermore, it is evident that PAP binding residues are mostly located inside, while sinigrin binding residues outside of the conserved regions (Fig. 3). Binding of the comparatively small substrate sinigrin induces only subtle structural changes. The Met186 may contribute to sinigrin coordination by forming a new hydrophobic contact with the substrate.

### Mutagenesis analysis of catalytically important residues

The residues Lys93, Thr96 and 97, Tyr130 and His155 are located in direct vicinity to the catalytic centre and may be involved in substrate proximity and orientation effects, proton transfer events, and the stabilization of transition state geometries, which could lower the energy barrier of chemical reaction (Table S1).

To test the importance of the five residues in the catalytic centre, we performed mutagenesis and enzymatic activity studies. Four of these amino acids are strictly conserved throughout the AtSOT family, indicating a significant function; Tyr130 is only partly conserved.

After mutating these residues to alanine, the mutants were tested with 3-methylthiopropyl Gl (3MTP), 8MTO and sinigrin as substrates (Table 1). In these assays, the activities of the mutants Lys93Ala, Thr97Ala, Tyr130Ala and His155Ala were below the detection limit. Thr96Ala showed residual activity with the preferred substrates 3MTP and 8MTO (12-fold reduction and 3-fold reduction, respectively), while with sinigrin no activity was detected (wild-type activity: 879 ± 410 pkatal mg^−1^).

### Enzyme kinetics and inhibition tests of AtSOT18

The AtSOT18 enzyme kinetics and the inhibition of the enzyme by PAP were analysed, as PAP is an important second messenger molecule in *A. thaliana* ^25–27^. In line with former studies^11, 14, 28^, the inhibition tests were performed with 3MTP instead of sinigrin. Product formation was detected by HPLC and used to determine the respective enzymatic activity at varying concentrations of donor and inhibitor molecules resulting in the two-dimensional titration data.

**Figure 6:**
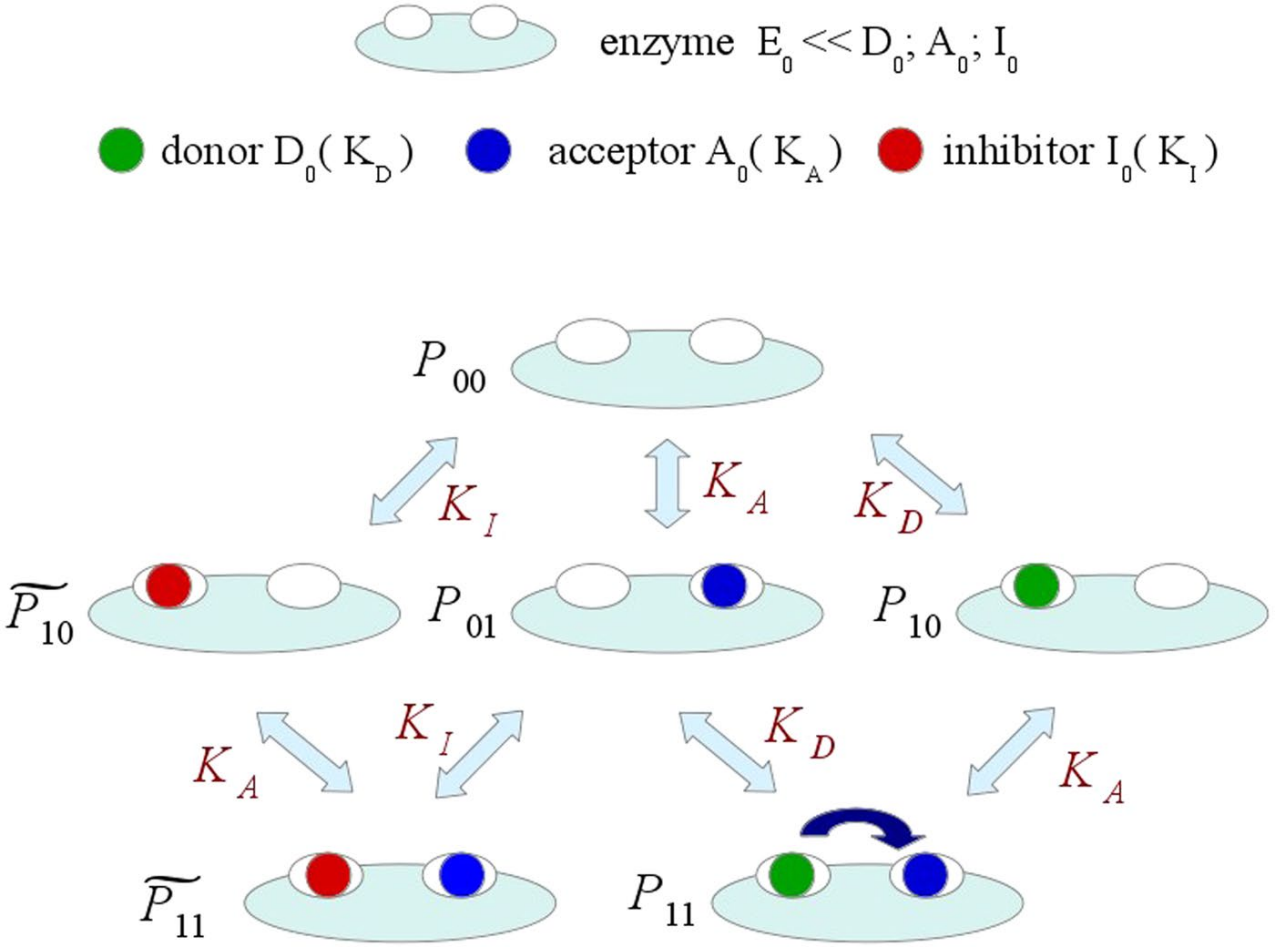
Model of the competitive inhibition of AtSOT18 by PAP. In equilibrium, six different populations of enzyme•ligand complexes are present with their respective probabilities *P*_*ij*_, where *i* and *j* represent the status of the donor and acceptor site, respectively. 0 and 1 represent non-occupied and occupied enzyme sites. ${\tilde{P}}_{ij}$ represents the probability of an enzyme•inhibitor complex species with the inhibitor bound to the donor site. *D*_*0*_, *A*_*0*_, and *I*_*0*_ represent the initial concentration of donor PAPS, acceptor ds-3MTP, and inhibitor PAP, respectively. Dissociation constants are displayed as *K*_*D*_ for the donor, *K*_*A*_ for the acceptor, and *K*_*I*_ for the inhibitor in equilibrium, respectively. The sulfuryl group transfer could only be catalysed within the state *P*_*11*_ where donor and acceptor site are each occupied with the substrates.

Analysis of these 2D enzyme kinetics data of AtSOT18 was based on the following assumptions: PAP and PAPS were expected to bind to the same site in the enzyme resulting in a competitive inhibition, as previously shown for other sulphotransferase enzymes^18, 20, 29^. This implies unaltered dissociation constants *K*_*D*_ for PAPS, *K*_*A*_ for 3MTP, and *K*_*I*_ for PAP. We also assumed that these three dissociation constants were independent of each other. Finally, the concentration of enzyme molecules *E*_*0*_ in the reaction was much lower than the initial ligand concentrations of donor *D*_*0*_, acceptor *A*_*0*_, and inhibitor *I*_*0*_. Therefore, the ligand concentrations at equilibrium were assumed to be unchanged as compared to the initial concentrations. Also, the correlation between the product 3MTP and the equilibrium population of AtSOT18•PAPS•ds-3MTP complex was assumed to be linear. In other words: once the enzyme had bound both ligand molecules, the reaction was catalysed, and two product molecules were released with respective dissociation rates. These assumptions result in a model with six equilibrium states depicted in Fig. 6. At pseudo-first order conditions (i.e. when *E*_*0*_ ≪ *A*_*0*_; *D*_*0*_; *I*_*0*_) the following system of algebraic equations represent the probabilities to find the enzyme in the respective occupied state:

1 $${P}_{00}+{\tilde{P}}_{10}+{P}_{10}+{P}_{01}+{\tilde{P}}_{11}+{P}_{11}=1$$

2 $$\frac{{P}_{10}}{{P}_{00}}=\frac{{P}_{11}}{{P}_{01}}=\frac{{D}_{0}}{{K}_{D}}$$

3 $$\frac{{P}_{11}}{{P}_{10}}=\frac{{P}_{01}}{{P}_{00}}=\frac{{\tilde{P}}_{11}}{{\tilde{P}}_{10}}=\frac{{A}_{0}}{{K}_{A}}$$

4 $$\frac{{\tilde{P}}_{10}}{{P}_{00}}=\frac{{\tilde{P}}_{11}}{{P}_{01}}=\frac{{I}_{0}}{{K}_{I}}$$

where:

5 $${P}_{ij}=\frac{{E}_{ij}}{{E}_{0}}$$

The resulting solution for the productive state *P*_*11*_ has the following hyperbolic form:

6 $${P}_{11}=\frac{{A}_{0}{D}_{0}}{{A}_{0}{D}_{0}+{A}_{0}{K}_{D}+{D}_{0}{K}_{A}+{K}_{D}{K}_{A}+{I}_{0}\frac{({A}_{0}{K}_{D}+{K}_{D}{K}_{A})}{{K}_{I}}}$$

**Figure 7:**
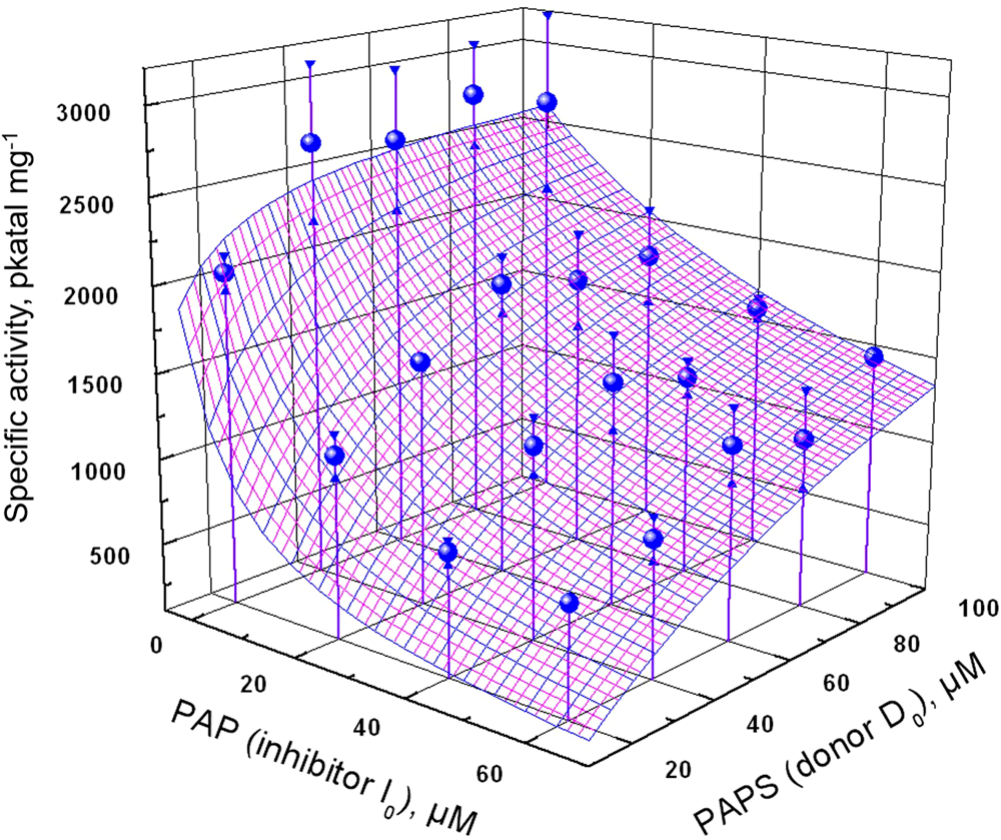
3D plot of AtSOT18 kinetic titration data their fit. Blue spheres represent the mean values of three replicates of the enzyme rate in pkatal mg^−1^. Triangles are the estimated errors of experimental points. The wire frame surface represents the fitted function. Ds-3MTP concentration was kept constant at 60 µM.

Our experimental data were therefore approximated by the function *V*_*max*_ *P_*11*_. Since the concentration of acceptor *A*_*0*_ was fixed at 60 µM and the *K*_*A*_ were equally set to 55 µM, based on conditions used in previous studies^11, 14, 28, 30^, this function contains three unknown parameters *K*_*D*_ *, K*_*I*_, and *V*_*max*_, and depends on the two independent variables: *D*_*0*_ and *I*_*0*_. For data analysis and non-linear least squares fit of multidimensional data the Igor Pro software was used (Wavemetrics, Lake Oswego, OR, USA). The best approximation of experimental data provides the following parameters for wild-type AtSOT18: *V*_*max*_ = 5200 ± 300 pkatal mg^−1^, *K*_*D *_ = 4.2 ± 2.6 µM, *K*_*I *_ = 3.0 ± 1.7 µM, with the RMSD value of 150 pkatal mg^−1^. The experimental data and fit are shown in Fig. 7.

The obtained kinetic data imply that turnover time of AtSOT18 reaction is close to 4 seconds. The difference in binding energies between PAP and PAPS is in the range of 10% of thermal fluctuation energy, which makes the PAP nucleotide moiety a major contributor to the PAPS binding energy.

### The source of ds-Gl SOT specificity

Another topic of interest was how the substrate specificity of ds-Gl SOTs is regulated at the molecular level. The major differences in substrate preference are between AtSOT18, which hardly accepts indolic ds-Gls, and AtSOT16, which highly prefers these^14^. To investigate structural differences between AtSOT18 and AtSOT16, a homology model of AtSOT16 was built based on our AtSOT18 structure. Comparison of the experimental AtSOT18 and homology AtSOT16 models revealed that both the amino-acid composition and the geometry of the Gl binding sites are highly conserved in both isoforms (Fig. S5). Therefore, we concluded that the substrate specificity of ds-Gl SOTs is most likely to be regulated by the residues outside of the active site pocket. Amino acid alignment suggested that the specificity may be provided by the three functional loops gating the entrance to the active site (Fig. S6). Loop 2, in particular, is highly heterogeneous, with only 30% of amino acids conserved. Loops 1 and 3 contain many non-conserved residues as well. In the absence of other selectivity sources directly in the active site or the adjacent regions, we hypothesize the loop regions to be most important for substrate specificity.

## Discussion

### Sulphotransferase biology

Several studies have investigated molecular and structural features of sulphotransferases, regarding the mechanism, specificity, and function, as reviewed recently^18, 20^. The focus was mainly on human SULTs^24^; but also SULTs from mice^21^, insects^31^ and prokaryotes^32^ were analysed in detail. In the plant kingdom, however, SOTs were mainly studied at a physiological level^10^, and until now, only one plant SOT structure was published, where the three substrate-binding loops showed no electron density at all^17^. Thus, molecular details of SOTs from plants and their structure-function relationship in comparison with other sulphotransferases remained unclear. Here, we demonstrated that the SOT18 structure from *A. thaliana* shares the classical fold of soluble eukaryotic SULT proteins, including conserved catalytic residues, PAPS binding region and flexible loops surrounding the substrate binding site (Fig. 2). Furthermore, the previously identified conserved motifs, such as 5′-PSB loop, 3′-PB loop and the P loop-like motif, which are involved in PAP- and PAPS binding could be identified^10^. Hence, it can be speculated that many general characteristics of sulphotransferases are also conserved in plants. A major difference between mammalian SULTs and plant SOTs seems to be the absence of a dimerization domain.

Analysing the ligand binding sites revealed that the PAP binding residues are mainly located in the regions I-IV that are conserved in all organisms (Fig. 3). High conservation of the PAPS binding site has been reported for previously solved structures from other organisms^18^ and could now also be confirmed for plant SOTs. Hence, it is plausible that residues responsible for the binding of various Gl substrates are located outside of these conserved regions. The high degree of binding site conservation for the sulphate donor on the one hand and very low conservation at the acceptor site on the other hand, illustrates how sulphotransferases can adapt to different substrates in various organisms. In mammals, for example, SULTs are involved in detoxification, thus sulphating a broad range of compounds. They also perform various specific tasks, like the homeostatic control of signalling molecules, such as oxysterols and steroids like dehydroepiandrosterone^20, 33^. In plants, they also fulfil a broad range of functions, some of general nature, such as AtSOT10 sulphating brassinosteroids^34^, and more specific functions such as the choline-*O*-sulphate SOT from *Limonium* as part of a highly specialized salt stress response^35^. Ds-Gl SOTs, found only within the plant order Brassicales, could be considered as a plant-specific SOT. In a previous study, we analysed ds-Gl SOTs in *Brassica napus* and identified a new subgroup of SOT18-like enzymes, which did not show any activity with the tested ds-Gls^28^. We speculated that due to genome triplication and allopolyploidization events in the evolutionary history from *A. thaliana* to *B. napus*, SOT18 might have undergone pseudo- (loss of function), or neofunctionalization (gain of new function). In the context of new structural data on AtSOT18, one could hypothesize that natural variation of the identified catalytic residues or residues involved in PAPS binding are likely to result in pseudo functionalization, while mutations in the substrate binding site and the flexible loops, could lead to neofunctionalization.

### Analysis of the catalytic centre

The high-resolution structure of the AtSOT18 complex provided detailed information about the spatial arrangement and conformation of the residues of the catalytic centre. Structural analysis suggests two possible functions for the active site residues: providing the proximity and orientation effect for the substrates and a direct impact on the catalytic reaction via charge transfer and/or stabilization of the transition state geometry. The five residues of the catalytic centre (Lys93, Thr96, Thr97, Tyr130, His155 (Fig. 4)) that stabilize the interface between the two substrates were tested by mutagenesis (Table 1). The dramatic effect of alanine mutations at all these positions on enzymatic activity confirmed the importance of these residues for catalysis.

**Table 1:** Mutational analysis of the AtSOT18 enzyme.

Activity in pkatal mg^−1^				
AtSOT18	3 MTP	8MTO	Sinigrin	I3M
Wild-type*	1624 ± 122	1618 ± 272	879 ± 410	501 ± 46
Lys93Ala	N.D.	N.D.	N.D.	—
Thr96Ala	122 ± 19	536 ± 73	N.D.	—
Thr97Ala	N.D.	N.D.	N.D.	—
Tyr130Ala	N.D.	N.D.	N.D.	—
His155Ala	N.D.	N.D.	N.D.	—
Pro136Ala	1726 ± 264	—	—	473 ± 18

Selected amino acids in the catalytic centre were mutated to alanine. The activity was tested with short-chained aliphatic 3 MTP, long-chained aliphatic Gl 8MTO, co-crystallized sinigrin and indolic Gl I3M. The 150 µL assays contained 80 mM Tris/HCl, pH 8.0, 9.2 mM MgCl_2_, 60 µM of the respective ds-Gl substrates, 1 µg purified protein, and 60 µM PAPS. The reactions were started by the addition of PAPS, incubated for 20 min at 37 °C, and stopped by incubation at 95 °C for 10 min. The formation of the respective sulphated product was analysed by HPLC at 229 nm. The specific activities are given in pkatal mg^−1^. N.D., not detectable; (—), not tested.

Based on these experiments and the structural conservation between AtSOT18 and human SULTs, we were able to adopt mechanistic information from human enzymes^19–21^. In AtSOT18, after charge neutralization of PAPS by conserved residues upon binding, His155 abstracts a proton from the ds-Gl and the PAPS sulphur is attacked by the newly formed nucleophile. For completion of the reaction, the partial participation of the nucleophile leads to a charge build-up on the bridging oxygen. This may facilitate the sulphate dissociation from PAP. The transition state could be stabilized by hydrogen bond formation between the sulphate oxygen of sinigrin and Thr96 or Tyr130 (Fig. 5a) as well as between the oxygen atoms of 5′-phosphate of PAP and Thr96 or Thr97 (Fig. 5b). Our structure presents one of the post-reactive species, a Michaelis product-complex. Interestingly, the sulphated sinigrin product has moved a bit compared to other sulphotransferase-ligand complexes.

High levels of amino-acid sequence and 3D structural conservation of the active sites of AtSOT18, 16, 12 and human SULT1A1 strongly indicate that the sulphotransferase catalytic mechanism is conserved between mammals and plants, thus confirming previous studies^17, 36^.

### Mechanism of PAPS binding

PAPS is a common substrate for all sulphotransferase isoforms. Our kinetic data indicate that the sulfuryl group of PAPS does not contribute significantly to the binding energy of the donor. At the same time, the structure of the AtSOT18•PAP•sinigrin complex reveals H-bond interactions of the substrate with Thr96, Tyr130 and His155 stabilizing the sulfuryl group in the active site. Our mutagenesis study showed that the disturbance of these interactions leads to a dramatic loss of enzymatic activity (Table 1). This seeming contradiction is resolved by our AtSOT18•PAP complex structure, where the oxygen positions of the sulphate moiety are occupied by the oxygen atoms of ethylene glycol (Fig. 4). The necessity to replace solvent oxygen atoms upon binding of the sulfuryl group leads to a near zero binding enthalpy balance, which explains the results of experimental kinetics analysis. The solvent oxygens in AtSOT18•PAP complex occupying the place of the sulfuryl group in the AtSOT18•PAP•sinigrin complex are very stable and have excellent electron density and low B-factors. The main contribution to the PAPS binding energy is thus provided by the PAP nucleotide moiety, which binds to a deep hydrophobic pocket between helices α3, α13 and α16, and the 3′-phosphate group.

The dissociation constants obtained from our two-dimensional titration experiment *K*_*D*_ (PAPS) = 4.2 ± 2.6 µM, *K*_*I*_ (PAP) = 3.0 ± 1.7 µM of AtSOT18 are relatively high compared to human SULTs. For human SULT2A1, 22 individual rate constants were estimated considering a dead-end-complex formation with PAP^29^. The dissociation constants determined by^29^ for PAPS were 0.2 µM and for PAP 0.3 µM. Furthermore, the K_*m*_ values for PAPS were determined for various other human SULTs and ranged between 0.07 µM and 1.6 µM^18^.

By transferring Gl synthesis genes into tobacco, thus enabling it to synthesize Gls, it could be shown that SOTs are not the bottleneck of synthesis. Instead, it was stated that the PAPS supply could be the limiting step for Gl synthesis^37^. A possible biological reason for the comparably low affinity of AtSOT18 for PAPS could be that there are three ds-Gl AtSOTs, hence a reduced affinity would preserve the limited PAPS pool. Since Gls are transported from the cytoplasm into the vacuole and are only biologically active upon cell disruption, Gl biosynthesis could be considered a foresighted safety mechanism and not a fast immediate stress response. Hence, the limited PAPS supply would be more available for SOTs that are involved in immediate stress response, such as AtSOT12 and AtSOT15 using salicylic acid and hydroxyjasmonate as substrates, respectively^38, 39^, if they have a lower K_*m*_ value for PAPS than AtSOT18. Respective kinetic data have not been determined for these SOTs yet.

Further to the regulative functions of PAPS, also the by-product of SOT reaction PAP is considered to be a retrograde signal for induction of stress response^25, 26^. PAP is suggested to move into the nucleus where it inhibits the RNA-degrading activity of 5′–3′ exoribonucleases, which leads to the prevention of post-transcriptional gene silencing of stress response genes. Further mutational studies of PAP catabolic genes led to the accumulation of ds-Gls and lower levels of Gls. It was suggested that this is either caused by inhibition of either PAPS transport or SOTs^40^. Here we could demonstrate that ds-Gl SOTs are indeed inhibited by PAP.

### The substrate specificity of ds-Gl SOTs

The substrate specificity of sulphotransferases in general, including the ds-Gl SOTs, is still hardly understood. Different members of plant or mammal sulphotransferase repertoires often have overlapping substrate spectra with each other, making it difficult to assign the enzyme′s specific function. Various attempts to group SOTs according to accepted substrates based on primary sequence analysis were unsuccessful^10, 20^. Comparison of the AtSOT18 Gl binding site with the one in a homology model of AtSOT16 showed no obvious differences that would explain the distinctions in substrate specificity. Also the extension of our search to the adjacent residues to the catalytic site could not explain the differences in the Gl binding affinities. Hence, we suggest that a specificity source of the ds-Gl SOTs might be provided by the non-conserved functional loops forming the Gl binding site, similar to the human enzymes^41–43^. However, substrate specificity cannot be entirely explained by the conformational properties of the gating loops. For example, for human SULT1A1 a molecular clamp mechanism was suggested, where two phenylalanine residues are repositioned in response to preferred substrates in such a way that stabilize the substrate′s phenolic residue in a catalytic enhancing position^44^. Even though we provided the first crystal structure of a plant SOT with bound ligands and complete electron density of the gating loops, further studies are needed to achieve a comprehensive understanding of the ds-Gl SOT selectivity mechanisms.

## Methods

### Expression, purification, and crystallization

The sequence encoding SOT18 from *Arabidopsis thaliana* (AtSOT18, At1g74090) was cloned into pQE-30 (Qiagen, Hilden, Germany) and expressed in *Escherichia coli* as described in^15^. Mutagenesis was performed as described by^11^. The purification of recombinant AtSOT18 protein by affinity chromatography was performed according to^15^ with modifications. An additional washing step with 0.12 M imidazole (20% buffer B + 80% buffer A; buffer B: 20 mM NaH_2_PO_4_, 0.5 M NaCl, 0.5 M imidazole, pH 7.4) was performed to obtain protein in a higher purity of up to 95%. The protein was dialysed in 20 mM Tris/HCl, pH 8.0 and 1 mM DTT for enzymatic assays or 20 mM HEPES, pH 8 for crystallization. Previously identified crystallization conditions (done by Prof. Dr. George N. Phillips, Jr., University of Wisconsin-Madison, USA) were used for further optimization by fine screens and additive screens. For the crystallization set-ups the concentrated protein sample was mixed gently with 4 mM PAP, and for the AtSOT18•PAP•sinigrin complex, with 4 mM PAP and 4 mM sinigrin. During the complex formation, the sample remained on the ice for 45 min, followed by a centrifugation step at 21,000 × g for 30 min. The fine screening was performed in 24-well plates for hanging and sitting drop plates with a total reservoir volume of 500 µL and 200 µL, respectively. The total droplet size was 1.8 to 2.2 µL; protein complex and reservoir solution were mixed in a ratio of 1:1. The additive screening was performed in a 96-well sitting drop plate by preparing the desired protein complex and reservoir solution, and mixing the reservoir with 10% volume of the Additive Screen HT™ - HR2-138 (Hampton Research, Aliso Viejo, USA).

Plate incubation and crystal growth documentation were performed using the Ministrel CrystalMation incubation and imaging system (Rigaku, Tokyo, Japan) for standard format plates (SBS format, Society for Biomolecular Screening). The measured crystals grew under 0.1 M 2-(N-morpholino) ethanesulfonic acid (MES) pH 5.9, 16% PEG_4000_, 160 mM NaCl, and 4% 1,3-butanediol (AtSOT18•PAP•sinigrin complex) and 0.1 M MES pH 5.9, 16.5% PEG_4000_, 160 mM NaCl and 5% 1-propanol (AtSOT18•PAP complex) at 18 °C.

### Diffraction data collection, structure determination and homology modeling

Before crystal harvest and freezing in liquid nitrogen the crystal was immersed in a cryoprotective solution (cryobuffer was identical to the reservoir conditions plus 20%(v/v) ethylene glycol). Diffraction data from the harvested crystals was collected at the European Synchrotron Radiation Facility at the beamline ID23-1. Crystallographic data and refinement statistics are summarized in Table 2. The structure was solved by Molecular Replacement using AMoRe program in the Collaborative Computational Project No. 4 (CCP4)^45^, with coordinates of an AtSOT12 (PDB ID 1Q44) as a starting model. Model building was performed using the Crystallographic object-oriented toolkit (COOT) V 0.7.2.1 and the CCP4 program suite V 6.3.0. For an automated overall refinement and electron density calculations, Refmac5 and ARP/warp Classic V 7.3.0 within the CCP4 package were used. Protein visualization and analysis were performed using Pymol and Schrödinger Maestro. Chemical equations were designed in ChemDraw. Homology models were created in Schrödinger Prime, using AtSOT18 as template.

**Table 2:** Crystallographic data and refinement statistics for the AtSOT18•PAP•sinigrin and the AtSOT18•PAP complexes.

	AtSOT18 complexed with	
	PAP, sinigrin	PAP, no acceptor
PDB code	5MEX	5MEK
**Crystal Parameters**		
space group	P 4_3_ 2_1_ 2	P 4_3_ 2_1_ 2
cell parameters		
*a*, *b*, *c* (Å)	63.82, 63.82, 209.97	62.74, 62.74, 201.5
*α*, *β*, *γ* (deg)	90, 90, 90	90, 90, 90
**Data collection**		
ESRF beamline	ID23-1	ID23-1
wavelength (Å)	0.91	0.91
Crystal mosaicity (deg)	0.066	0.073
Wilson *B*-factor	37.3	32.7
resolution range (Å; total/high)	19.95–1.92/2.02–1.92	19.99–1.74/1.84–1.74
unique reflections (total/high)	34,239/4625	42,500/6394
completeness (total/high) %	99.63/98.3	99.9/100.0
〈*I*/σ(*I*)〉 (total/high)	22.68/3.21	20.44/2.65
*R*_*sigma*_ (total/high) %	2.7/31.4	2.8/37.4
*R*_*int*_ (total/high) %	6.5/54.4	9.3/75.3
**Refinement statistics**		
included amino acids	26–347	26–347
number of protein atoms	2652	2698
number of H_2_O molecules	286	242
*R*_*work*_/*R*_*free*_ %	18.3/22.5	18.1/21.5
RMS deviation for bonds (Å)/angles (deg)	0.02/2.047	0.022/2.145

Information on high-resolution data referred to the outer 0.1 Å of the resolution shell.

### Preparation of substrates

The ds forms of the parent Gl derived from methionine and tryptophan were prepared as described by^46^. The following Gl were used in the experiments in their ds forms: 3 MTP (glucoiberverin) from *Erysimum pumillum*, 8MTO from *Arabis stelleri*, I3M (glucobrassicin) from *Isatis tinctoria*. 2-Propenyl Gl (sinigrin) and PAP was bought commercially (Sigma-Aldrich, Taufkirchen, Germany). PAPS was obtained from Prof. H. R. Glatt, Institute of Human Nutrition, Berholz-Rehbruecke, Germany.

### Enzyme activity measurements and analysis of the kinetic parameters

Enzymatic AtSOT18 assays were performed as described in^28^. For inhibition experiments, a set of two-dimensional titration experiments was performed to determine the dissociation constants *K*_*D*_ of the donor PAPS and the *K*_*I*_ of the competitive inhibitor PAP. In the experiments, the amounts of enzyme AtSOT18 and acceptor ds-3MTP were kept constant at 0.5 µg and 60 µM, respectively. The concentration of the sulphate donor PAPS was varied to 20, 40, 60, 80, and 100 µM and the inhibitor PAP was added at concentrations of 0, 20, 40, and 60 µM. Each reaction was prepared in triplicate. The two-dimensional non-linear least squares fit of the measured data with donor PAPS and inhibitor PAP as variables were performed. IgorPro V4.00 (WaveMetrics Inc., Lake Oswego, USA) was used for nonlinear least squared Levenberg-Marquard fitting of the two-dimensional experimental data (x as inhibitor concentration I_0_ and y as donor concentration D_0_) to the theoretical hyperbolic function derived from the model assumptions. The dissociation constants for the donor PAPS *K*_*D*_, and the inhibitor PAP *K*_*I*_ and *V*_*max*_ were determined in an iterative minimization of the RMSD to the measured data. The dissociation constant *K*_*A*_ and concentration *A*_*0*_ of the acceptor ds-3MTP was set to 55 µM according to literature^14^ and 60 µM due to the experimental procedure.

## Electronic supplementary material

Dataset 1
