# Supplementary material for: Structural and biochemical studies of sulphotransferase 18 from Arabidopsis thaliana explain its substrate specificity and reaction mechanism
Source: Sci Rep. 2017 Jun 23;7:4160. doi: 10.1038/s41598-017-04539-2 (PMC5482895; doi:10.1038/s41598-017-04539-2)
Supplement: Supplementary file 1 — Dataset 1 [file 41598_2017_4539_MOESM1_ESM.doc]

**Structural and biochemical studies of sulphotransferase 18 from *Arabidopsis thaliana* explain its substrate specificity and reaction mechanism**

Felix Hirschmann^1^, Florian Krause^1^, Petra Baruch^2,3^, Igor Chizhov^2,3^, Jonathan Wolf Mueller^4,5^, Dietmar J. Manstein^2,3^, Jutta Papenbrock^1^,*, and Roman Fedorov^2,3^*

**Supplementary data**

**Table S1. Summary of the amino acids putatively involved in sinigrin and PAP binding, as well as catalysis.**

| **Proposed residue** | **Possible role of the amino acid** |
| --- | --- |
| E54 | substrate binding (dsG1) |
| E193 | substrate binding (dsG1) |
| R51 | substrate binding (dsG1) |
| Y306 | substrate binding (dsG1) |
| L190 | substrate binding (dsG1) |
| F189 | substrate binding (dsG1) |
| M186 | substrate binding (dsG1) |
| Y311 | substrate binding (dsG1) |
| V305 | substrate binding (dsG1) |
| L65 | substrate binding (dsG1) |
| S6 | substrate binding (dsG1) |
| L68 | substrate binding (dsG1) |
| I133 | substrate binding (dsG1) |
|  |  |
| K93 | stabilization of transition state, proton or electron transfer |
| Y130 | stabilization of transition state, proton or electron transfer |
| H155 | stabilization of transition state, proton or electron transfer |
| T97 | stabilization of transition state, proton or electron transfer |
| T96 | stabilization of transition state, proton or electron transfer |
|  |  |
| R177 | substrate binding (PAPS), proton or electron transfer |
| S185 | substrate binding (PAPS), proton or electron transfer |
| R313 | substrate binding (PAPS), proton or electron transfer |
| F284 | substrate binding (PAPS) |
| R247 | substrate binding (PAPS), proton or electron transfer |
| W98 | substrate binding (PAPS) |
| Y243 | substrate binding (PAPS), proton or electron transfer |

**
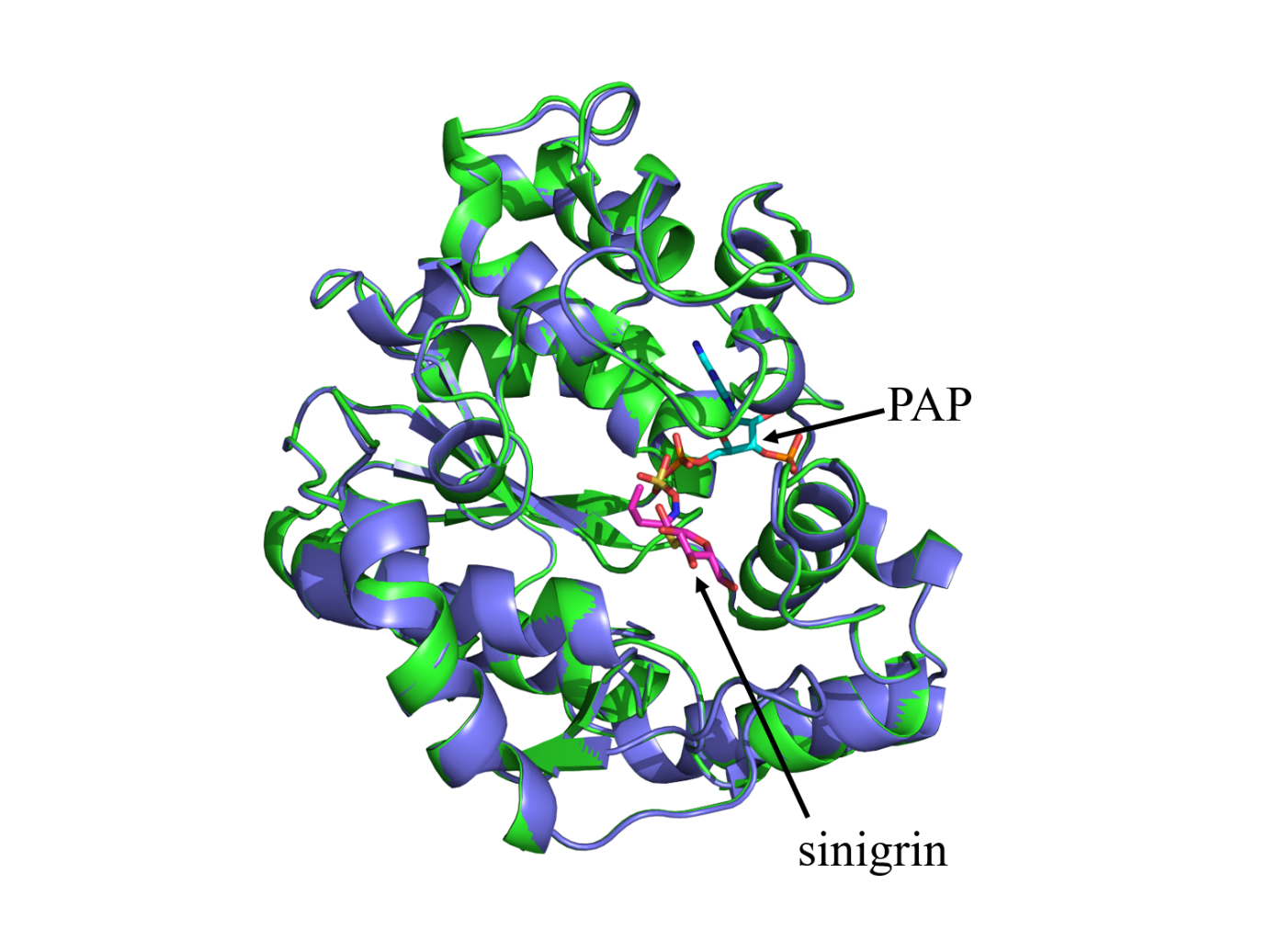
Figure S1. The superimposition of the overall structures** shows that there are no major differences between the two solved structures of AtSOT18•sinigrin•PAP (green) and AtSOT18•PAP (cyan). The structures have been superimposed by structural alignment of the complete structures in Pymol.

**
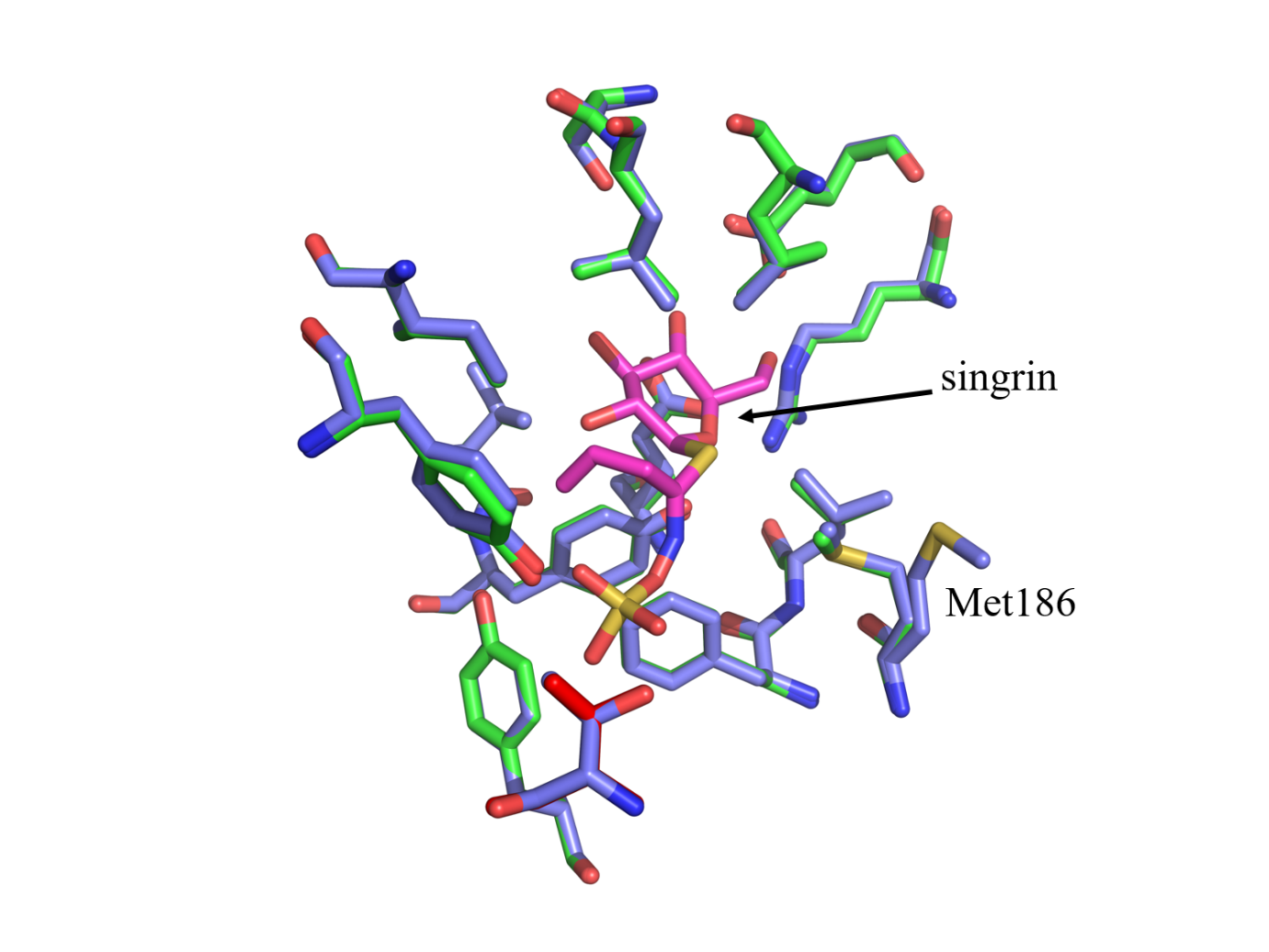
Figure S2. Comparison of the AtSOT18•PAP•sinigrin (green) and AtSOT18•PAP (blue) complex’ sinigrin (magenta) binding site**. All sinigrin surrounding residues are in both complexes in the same position, except for Met186, which shows a double confirmation in the AtSOT18•PAP (blue) complex. In one conformation the methionine side chain is oriented towards the acceptor, providing an additional stabilization to its hydrophobic moiety.

**
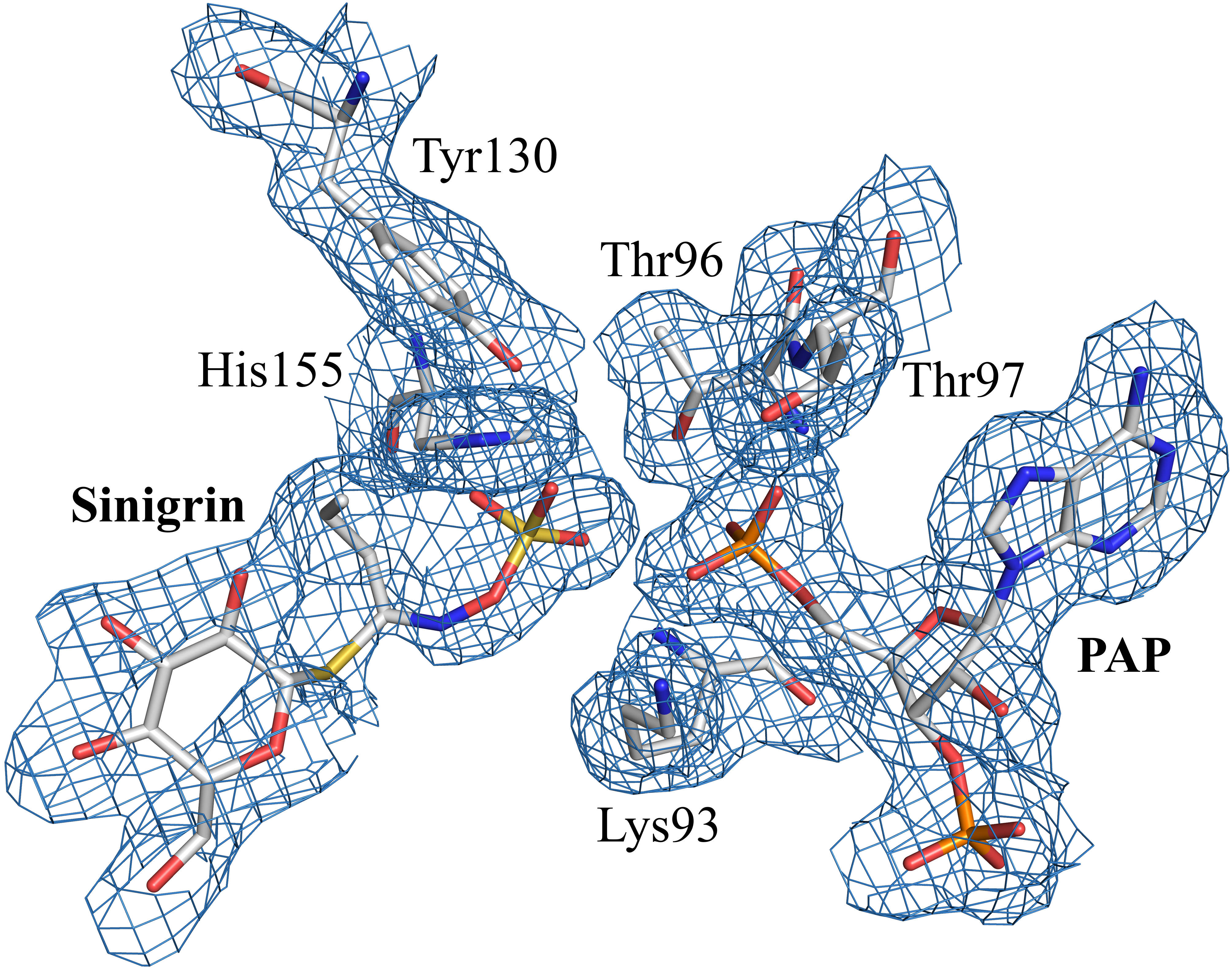
**

**Figure S3.** Electron density omit map around the ligands and the active site residues in the AtSOT18•PAP•sinigrin complex structure, contoured at 0.8 σ level.

**
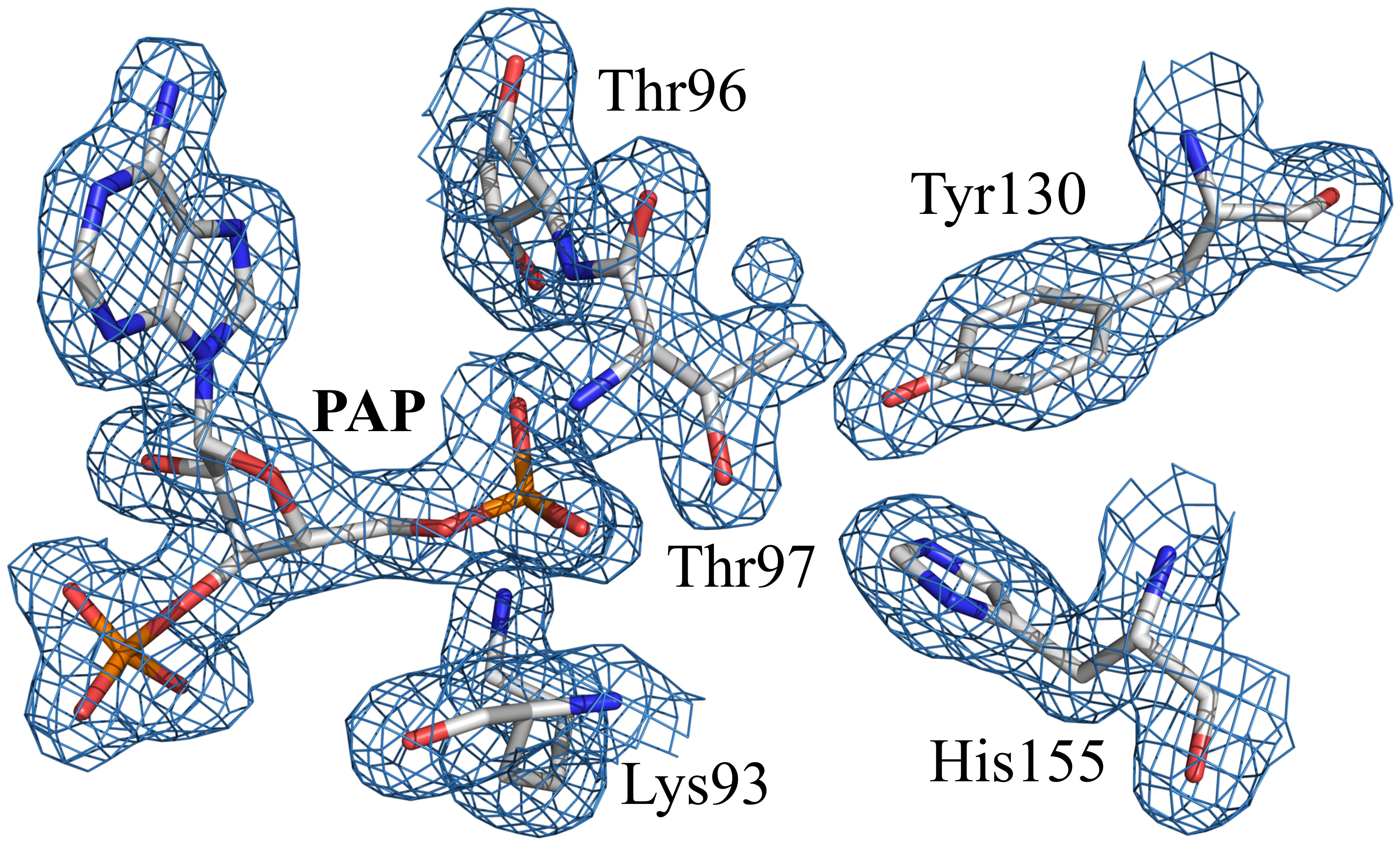
Figure S4.** *2F_o_ – F_c_*electron density omit map around the ligands and the active site residues in the AtSOT18•PAP complex structure, contoured at 1σ level. The quality of the electron density allowed an exact determination of the position and stereochemistry of the PAP.


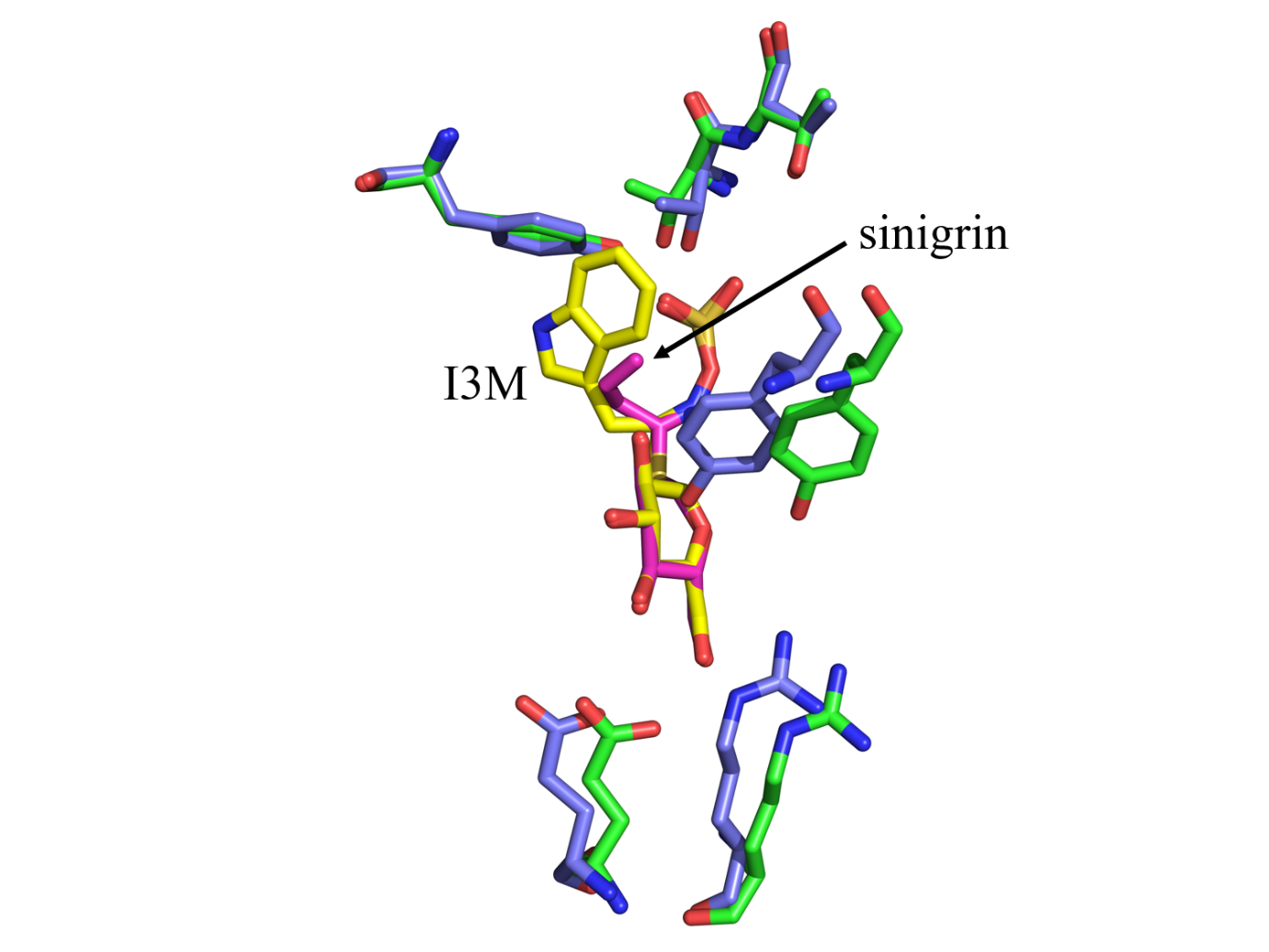


**Figure S5.** Structural alignment of the sinigrin (magenta) binding residues of the AtSOT18•PAP•sinigrin (green) and AtSOT16 homology model (cyan) with superimposed indol-3-ylmethylglucosinolate (I3M). Comparison of the experimental AtSOT18 and homology AtSOT16 models revealed that both the amino-acid composition and the geometry of the Gl binding sites are highly conserved in both proteins.


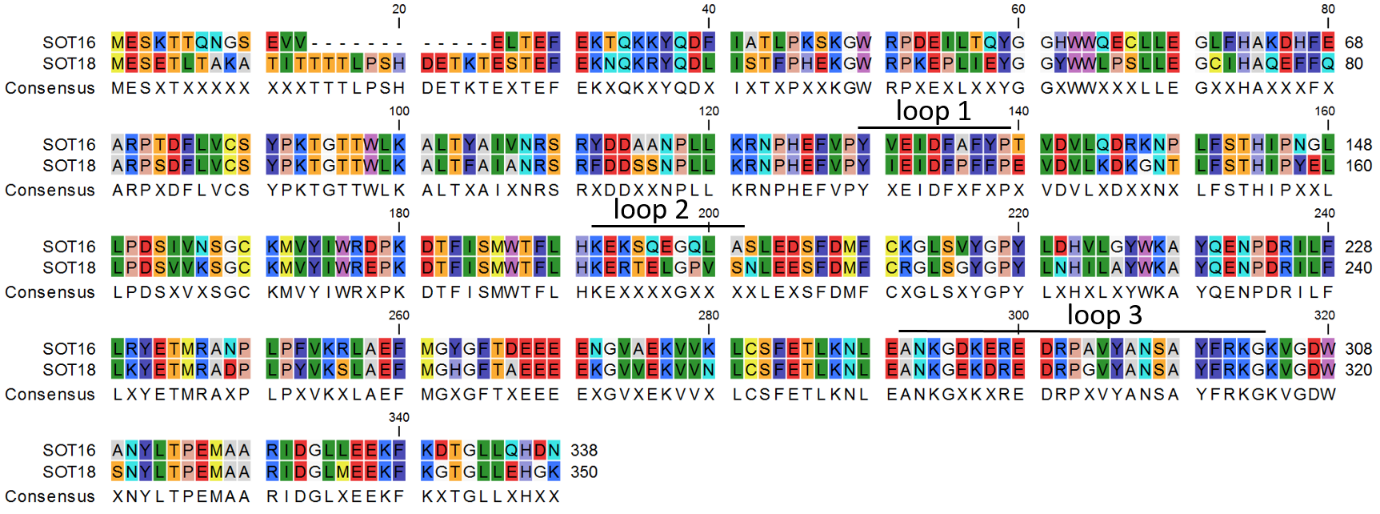


**Figure S6. Alignment of the AtSOT16 and AtSOT18 protein sequences.** The three flexible loops forming the ds-Gl binding site are highlighted. Loop 2 in particular is highly heterogeneous, with 30% of amino acids conserved. Loops 1 and 3 also contain many non-conserved residues.
